# Age differences in demographic and clinical characteristics among veterans with chronic low back pain: a cross-sectional study of baseline findings from the Veteran Response to Dosage in Chiropractic Therapy (VERDICT) trial

**DOI:** 10.1186/s12998-025-00613-z

**Published:** 2025-10-13

**Authors:** Stacie A. Salsbury, Cynthia R. Long, Jacob McCarey, Anthony J. Lisi, Anna Steward, Robert B. Wallace, Christine M. Goertz

**Affiliations:** 1 Palmer Center for Chiropractic Research, Palmer College of Chiropractic, 741 Brady Street, Davenport, IA 52803 USA; 2 VA Connecticut Healthcare System, 950 Campbell Avenue, West Haven, CT 06516 USA; 3 College of Public Health, The University of Iowa, 145 Riverside Dr, Iowa City, IA 52242 USA; 4 Duke University School of Medicine, 300 W Morgan St, Durham, NC 27701 USA

**Keywords:** Veterans, Low back pain, Chronic pain, Musculoskeletal pain, Mental health, Factors, Demographic, Prescriptions, Pragmatic clinical trial, Age factors, Manipulation, Chiropractic

## Abstract

**Background:**

Veteran Response to Dosage in Chiropractic Therapy (VERDICT) was a pragmatic randomized trial testing chiropractic dosage effects in 766 veterans with chronic low back pain (CLBP) of ≥ 3 months. This cross-sectional analysis compares baseline characteristics of younger (18-to-64 years) and older veterans (≥ 65 years).

**Methods:**

Data were collected from February 22, 2021 to May 21, 2025 via electronic health records and REDCap questionnaires. Descriptive statistics and tests of group differences were performed using SAS.

**Results:**

VERDICT enrolled 188 older veterans (25%; mean 72 years) and 578 younger veterans (75%; mean 44 years). More female (24.7% vs. 10.6%, *p* < .001), Black (18.9% vs. 12.2%), and Hispanic (11.8% vs. 3.7%, *p* = .001) veterans comprised the younger cohort. Employment differed (*p* < .001) with older veterans retired (78.2% vs. 14.2%) and younger veterans employed (59% vs. 16.5%). About 14% lived rurally and period of military service was similar. Pain profiles were similar between younger and older veterans for > 5 years duration (78.4% vs. 73.4%), high-impact chronic pain (64.5% vs. 62.2%), mean pain interference [63.8(4.8) vs. 63.2(5.0)], and mean back-related disability (primary outcome) [11.9(5.2) vs. 13.3(4.9)]. Younger veterans scored significantly higher than older veterans for depression (44.8% vs. 31.4%, *p* = .001), anxiety (41.5% vs. 20.7%, *p* < .001), post-traumatic stress (38.4% vs. 17.6%, *p* < .001), sleep disturbance (57.1% vs. 34.6%, *p* < .001), and high-risk alcohol use (25.4% vs. 18.1%, *p* = .05). Previous chiropractic use was similar (younger 75.4% vs. older 80.3%). Medications in past 3 months differed with younger veterans reporting cannabis (25.8% vs. 12.8%, *p* < .001) and muscle relaxants (31.7% vs. 17.6%, *p* < .001) and more older veterans reporting acetaminophen (63.3% vs. 49.3%, *p* < .001) and gabapentin (34% vs. 20.1%, *p* < .001). NSAIDs use was highest among both younger (62.8%) and older (56.9%) veterans. While two-thirds had tried exercise in the past 3 months, only 16% reported exercising for their pain condition, with older veterans more likely to report providers encouraging physical activity.

**Conclusions:**

Similar pain profiles were reported among older and younger veterans seeking chiropractic care for CLBP within a clinical trial. However, potentially important age differences were noted in demographics, mental health and substance use, and CLBP treatments.

**Trial registration:**

ClinicalTrials.gov: NCT04087291. Date of Registration: 9/12/2019. Enrollment Duration: 2/22/2021 (first participant enrolled) through 5/10/2024 (last participant enrolled).

**Supplementary Information:**

The online version contains supplementary material available at 10.1186/s12998-025-00613-z.

## Background

Chronic pain affects 20% of adults in the United States (U.S.), with high impact chronic pain (HICP) severely limiting daily activities for 6.9% of the population [1]. Military veterans experience a higher prevalence of chronic pain (27.5%) compared to non-veterans (19.2%), as well as HICP (11.6% vs. 6.5%) [1]. Veterans who receive healthcare through the Veterans Health Administration (VHA) are more likely to report chronic pain than veterans who receive treatment elsewhere [2]. Among chronic pain conditions, chronic low back pain (CLBP) is reported by 40% of veterans compared to 30% of non-veterans [3]. Veterans with CLBP face many health-related challenges when managing their pain, often arising from their complex medical conditions (such as cardiovascular disease, diabetes, depression, anxiety, and post-traumatic stress disorder), medication use and polypharmacy to treat those conditions, and psychosocial factors (alcohol, tobacco, and substance use; varying employment and living arrangements) [4, 5]. 

These statistics underscore the urgent need to develop effective self-care and healthcare solutions for veterans seeking pain management services, particularly those navigating the organizational complexities of the Veterans Affairs (VA) healthcare system [6, 7]. Such work is important since the VA is the largest integrated healthcare system in the U.S., serving over 9 million enrollees at nearly 1,300 facilities [6]. The VA, in collaboration with the Department of Defense (DOD), has moved in this direction by developing the *VA/DOD Clinical Practice Guideline for the Diagnosis and Treatment of Low Back Pain* [8]. The guideline offers evidence-based recommendations for the care of VA patients, many of which are consistent with the non-pharmacological, multimodal care offered by doctors of chiropractic practicing within VA [9–11]. These recommendations include high quality services such as chiropractic manipulative therapy, clinician-directed exercise, and self-care education [11]. However, published utilization rates for VA chiropractic care is relatively low, with about 3.5% of veterans overall accessing these services, which include 2% who receive community care only, 1.3% on-station care only, and 0.2% both care locations [12]. Projections suggest that overall utilization of VA chiropractic services could reach 8.9% by FY2027 [12]. This will require additional information about the health needs of veterans who use VA chiropractic care.

Chiropractic care can improve clinical outcomes for people with CLBP, including older male veterans [13], although evidence is lacking in other VA populations. Patients receiving chiropractic care for spinal pain, including veterans, are less likely to fill opioid prescriptions [14–16]. Moreover, a study analyzing Medicare claims data from older adults aged 65 years and older found the use of spinal manipulation led to fewer escalations in care when compared to opioid analgesic therapy [17]. Despite these benefits, older adults tend to utilize chiropractic care less frequently than their younger and middle-aged counterparts due to barriers such as limited access, lack of awareness, and misconceptions about its effectiveness [2, 5]. Historically, veterans seeking chiropractic care report persistent musculoskeletal issues, particularly LBP, and have an average age in their mid-50s [15, 18]. Recent trends demonstrate higher percentages of veterans under age 65 years [19] and more female veterans using chiropractic care [20, 21]. These changing patient demographics within VA highlight the need for a study exploring differences in socioeconomic background and clinical conditions among older and younger veterans seeking chiropractic care.

The Veteran Response to Dosage in Chiropractic Therapy (VERDICT) trial was a large-scale, randomized, pragmatic clinical trial (PCT) [22] conducted as part of the Pain Management Collaboratory (PMC), a network of 16 PCTs and a coordinating center charged with studying the effectiveness and implementation of non-pharmacological interventions for pain and co-occurring conditions in veteran and military healthcare systems [23–25]. The VERDICT trial was designed to evaluate the effectiveness of varied dosage levels of chiropractic care for the management of CLBP in veterans [22]. One aim of the PMC trials is to better understand the biopsychosocial experience and healthcare needs of veterans with chronic pain among key patient populations, including younger and older veterans [26]. The purpose of this study was to compare age differences in the baseline demographic and clinical characteristics of younger (< 65 years) and older (≥ 65 years) veterans who participated in the VERDICT trial.

## Methods

### Study design

This cross-sectional study is a secondary analysis of baseline data from VERDICT, a pragmatic, parallel group, multisite randomized clinical trial designed to identify the effectiveness of different dosages of chiropractic care for veterans with CLBP [22]. The VERDICT trial was conducted at 4 VA Healthcare Systems: Iowa City, Iowa; West Haven, Connecticut; Minneapolis, Minnesota; and Greater Los Angeles, California. The study received ethics approval from the Yale University Institutional Review Board (IRB) (Yale IRB 2000025406; 5/22/2019) and the VA Central IRB (VA CIRB 1613761; 18–34; 1/19/2019). VERDICT was registered prospectively at ClinicalTrials.gov (NCT04087291). The first participant was enrolled on 2/22/2021, the final participant was enrolled on 5/10/2024, which constituted the final date of baseline data collection. Clinical trial data were collected through May 21, 2025. This manuscript was prepared using the STROBE reporting checklist [27]. 

### Participants

VERDICT enrolled 766 participants who currently used VA healthcare services and met these inclusion criteria: age ≥ 18 years; self-reported CLBP [LBP that persisted for ≥ 3 months with pain on at least half the days in the last 6 months]; LBP pain intensity score ≥ 2 at baseline on the Pain, Enjoyment and General Activity (PEG) instrument [28]; LBP disability score ≥ 4 at baseline on the Roland Morris Disability Questionnaire (RMDQ); ability to comprehend study details without a proxy; an International Classification of Diseases, Tenth Revision (ICD-10) [29] diagnosis confirming neuromusculoskeletal LBP [see Supplemental Materials]; and willingness to attend chiropractic visits for up to 1 year [29, 30]. Participants were deemed ineligible if they met any exclusion criterion: inability to complete outcomes or provide informed consent; plans to move; currently under chiropractic care; no phone and/or no email address; participating in another study of pain treatments; current or planned hospice care; or current or planned pregnancy. As this was a pragmatic trial, our goal was to reflect routine chiropractic care in VA settings [22]. Thus, participants were excluded for clinical conditions only if the licensed Doctor of Chiropractic who completed a clinical examination determined that chiropractic care could not be safely provided due to suspected or confirmed conditions, such as neurological deficits, visceral pathology, fractures, infections, or inflammatory arthropathies [22]. The age cutoff of 65 years was selected for this analysis as veterans in this age group may receive healthcare funding from both the VA and the Medicare program, leading to the potential for duplicative payments for some services [31]. 

### Data collection

Our data collection methods are described in detail in the published study protocol [22]. Demographic data, service-connected disability status, period of military service, national and state deprivation indexes, and ICD-10 codes for LBP were obtained from the VA electronic health record (EHR). Patient-reported data were collected via survey at the baseline visit using Research Electronic Data Capture (REDCap; Vanderbilt University, Nashville, Tennessee, U.S.) [32]. The primary outcome was the RMDQ score [30, 33]. Secondary outcomes included pain intensity and overall pain interference via the PEG instrument and the Patient-Reported Outcomes Measurement Information System (PROMIS^®^) domains of pain interference, sleep disturbance, fatigue, satisfaction with roles and activities, self-efficacy, and global physical and mental health that were collected using computerized adaptive testing [28, 34]. We used standardized instruments to assess alcohol use, depression, anxiety, post-traumatic stress disorder (PTSD), and sleep disturbance [34–40]. The dichotomization thresholds for these instruments are well-supported by current clinical guidelines and validation studies in veteran and primary care populations and were harmonized to allow comparison of core outcomes measures across trials aligned with the PMC [24].

- Alcohol Use: Alcohol Use Disorders Identification Test (AUDIT-C) - Scores were dichotomized using U.S. Preventive Services Task Force and VA/DoD thresholds (≥ 4 for men, 0.86 sensitivity, 0.72 specificity; ≥3 for women, 0.6 sensitivity, 0.96 specificity), which have demonstrated acceptable detection of unhealthy alcohol use in primary care and military/veteran population [41–43].
- Depression: The Patient Health Questionnaire (PHQ-2), with a cut-off score of ≥ 3, which is considered a positive screen for depression in VA settings (0.97 sensitivity, 0.91 specificity) [44, 45].
- Anxiety: The Generalized Anxiety Disorder (GAD-7) scale used a threshold score of ≥ 10 (0.89 sensitivity, 0.91 specificity) [36].
- Post-Traumatic Stress Disorder: The PTSD checklist for DSM-5 (PCL-5) used a threshold score of ≥ 33 (0.87 sensitivity, 0.91 specificity) [46].
- Sleep Disturbance (PROMIS-SD): The PROMIS^®^ sleep disturbance measure used a T-score ≥ 60 to assess sleep problems in persons with mental health comorbidities [38].

Participants also self-reported their use of medications, cannabis, previous chiropractic care, and exercise for LBP. To determine rurality, participants’ zip codes were matched to Rural-Urban Commuting Area (RUCA) codes from the 2010 U.S. Census, where RUCA Code 1 was classified as Urban/Metropolitan and RUCA Codes 2–10 were classified as rural (codes 2 to 3 metropolitan commuting, codes 4 to 6 micropolitan, codes 7 to 9 small town, and code 10 rural area) [47]. The area deprivation index (ADI) is a ranking of U.S. neighborhood at the zip code level of socioeconomic disadvantage based on education, employment, housing-quality and poverty measures [48]. Higher ADI national percentiles or state deciles indicate higher socioeconomic disadvantage.

### Data analysis

VERDICT had very little missing baseline data. Many variables, including age and other demographic data, were complete for all 766 participants. Variables with some missing data were missing for less than 1–2% of participants. Therefore, we did not utilize imputation or any other techniques for handling missing data. Descriptive statistics characterized VERDICT participants grouped by age (older adults: ≥65 years; younger adults: <65 years) at baseline. Mean and standard deviation (SD) are presented for continuous variables, while counts and percentages are presented for categorical variables. PROMIS T-scores are constructed such that a referent population has a mean of 50 and a standard deviation of 10. Group differences in categorical variables between younger and older VERDICT participants were assessed using Chi-square tests. Differences in numeric variables between younger and older VERDICT participants were assessed using 2-sided, 2-sample t-tests, and results are presented as mean differences with 95% confidence intervals. All analyses were performed using SAS (Release 9.4; SAS Institute Inc., Cary, North Carolina).

## Results

### Demographic characteristics

Supplemental material provides enrollment information and demographic characteristics by clinical site. Table 1 compares the demographic characteristics of younger (< 65 years, *n* = 578, 75%) and older veterans (≥ 65 years, *n* = 188, 25%) enrolled in VERDICT. While the overall mean age of participants was 51 years (range 22 to 87), a considerable age gap was noted between the older veterans who had a mean age of 72 years and the younger veterans who had a mean age of 44. Older veterans were more likely than younger veterans to indicate male sex (89% vs. 75%) and White racial background (78.2% vs. 66.6%). Higher percentages of female (24.7% vs. 10.6%, *p* < .001), Black (18.9% vs. 12.2%), and Hispanic (11.8% vs. 3.7%, *p* = .001) veterans were enrolled in the younger cohort, as is consistent with changing demographics within the U.S. military population.

**Table 1:** Baseline demographic characteristics of younger and older veterans enrolled in the VERDICT trial (*n* = 766)

	Age < 65 years	Age ≥ 65 years	*p* value
Participants enrolled, n (%)	578 (75)	188 (25)	
Age, mean (range)	44 (22–64)	72 (65–87)	
Sex at birth, n (%)			*p* < .001
Male	435 (75.3)	168 (89.4)	
Female	143 (24.7)	20 (10.6)	
Race, n (%)			*p* = .03
White	385 (66.6)	147 (78.2)	
Black or African American	108 (18.7)	23 (12.2)	
Asian	11 (1.9)	1 (0.5)	
American Indian or Alaskan Native	2 (0.4)	4 (2.1)	
Native Hawaiian or Pacific Islander	7 (1.2)	1 (0.0)	
Multiracial	8 (1.4)	0 (0.0)	
Unknown or not reported	57 (9.9)	12 (6.4)	
Ethnicity, Hispanic or Latino, n (%)	68 (11.8)	8 (4.3)	*p* = .002
Highest level of education, n (%)			*p* = .44
Some grade school or high school	4 (0.7)	6 (3.2)	
High school graduate	90 (15.6)	29 (15.4)	
General Education Development (GED) or equivalent	12 (2.1)	5 (2.7)	
Some college or other program, no degree	157 (27.2)	50 (26.6)	
Associate degree: occupational, technical, vocational, or academic	107 (18.5)	38 (20.2)	
Bachelor’s degree (BA, AB, BS, BBA)	130 (22.5)	35 (18.6)	
Master’s degree (MA, MS, MEng, MEd, MBA)	68 (11.8)	23 (12.2)	
Professional degree (MD, DC, DDS, DO)	3 (0.5)	1 (0.5)	
Doctoral degree (PhD, EdD)	6 (1.0)	1 (0.5)	
Military service period, n (%)*			*p* = .42
Vietnam Era	112 (19.6)	32 (16.6)	
Post-Vietnam Era	88 (15.4)	24 (12.4)	
Persian Gulf War	372 (64.9)	136 (70.5)	
Other or none	1 (0.2)	1 (0.5)	
Employment status, n (%)			*p* < .001
Working for pay	341 (59.0)	31 (16.5)	
Retired	82 (14.2)	147 (78.2)	
Not currently employed	90 (15.6)	5 (2.7)	
Taking care of house or family	8 (1.4)	1 (0.5)	
Other	56 (9.7)	4 (2.1)	
Percent service-connected disability status n (%)^#^			*p* < .001
0	5 (0.9)	4 (2.1)	
1–25	34 (5.9)	26 (13.8)	
26–50	51 (8.8)	14 (7.5)	
51–75	82 (14.2)	21 (11.2)	
76–100	344 (59.5)	66 (35.1)	
Unknown	62 (10.7)	57 (30.3)	
Relationship status, n (%)			*p* < .001
Married or living with partner	282 (48.8)	105 (55.9)	
Divorced or separated	137 (23.7)	43 (22.9)	
Widowed	6 (1.0)	22 (11.7)	
Never been married	153 (26.5)	18 (9.6)	
Neighborhood status, n (%)			
Rurality	82 (14.2)	26 (13.8)	*p* = .90
Area deprivation index, national, n (%)			*p* = .27
< 80	449 (77.7)	142 (75.5)	
80–84	21 (3.6)	3 (1.6)	
≥ 85	28 (4.8)	14 (7.5)	
Unknown	80 (13.8)	29 (15.4)	
Area deprivation index, state, n (%)			*p* = .29
< 5	214 (37.0)	75 (39.9)	
5–7	174 (30.1)	43 (22.9)	
≥ 8	110 (19.0)	41 (21.8)	
Unknown	80 (13.8)	29 (15.4)	

*Military service period data differs from other baseline variables as these data were pulled from the electronic health record at a later timeframe. #In the U.S. Department of Veterans Affairs (VA) system, service-connected disability ratings reflect the extent to which military-related conditions affect a veteran’s daily functioning and employability. Ratings range from 0% to 100% in 10% increments and determine eligibility for compensation and VA benefits. Combined ratings for multiple conditions are calculated using a standardized formula, not simple addition. Ratings of 50% or higher confer additional benefits, including priority health care access [97]

Educational status did not differ between age cohorts. Relationship status differed (*p* < .001) with more older veterans indicating widow status (11.7% vs. 1%) and more younger veterans reporting never being married (26.5% vs. 9.6%). Employment status differed (*p* < .001) with more older veterans retired (78.2% vs. 14.2%) and more younger veterans currently working for pay (59% vs. 16.5%). Younger veterans were more likely than older veterans to report not being currently employed for a reason other than retirement (15.6% vs. 2.7%) or to indicate some other type of employment situation (9.7% vs. 2.1%). Service-connected disability also differed, with 59.5% of younger veterans ranked as 76–100% disabled compared to 35.1% for older veterans. Around 14% of veterans in each age group lived in rural settings, with the Iowa City site reporting the highest percentage of rural veterans (26.4%) and Los Angeles the lowest (3.8%). ADI did not differ between younger and older veterans at the National or State level yet indicated that many veterans lived in neighborhoods that are socioeconomically disadvantaged.

## Diagnoses, pain quality, and functional status characteristics

Table 2 reports the complex LBP diagnoses, high intensity and long duration of CLBP, and widespread functional impairments among VERDICT participants at baseline. ICD-10 codes did not differ between age groups. General mechanical LBP was similar between younger and older veterans (89.6% vs. 87.8%), as was radicular LBP (18.0% vs. 21.3%). Younger (78.4%) and older veterans (73.4%) reported CLBP lasting > 5 years as well as high levels of high-impact chronic pain (64.5% vs. 62.2%). Older veterans reported a slightly higher LBP disability at baseline than younger veterans (mean RMDQ difference 1.4, 95% confidence interval 0.6 to 2.3), with scores in both groups indicating moderate physical disability. Younger and older veterans had moderate average pain intensity (mean PEG pain intensity 6.2 vs. 5.9) and pain interference (mean PROMIS pain interference 63.8 vs. 63.2). Older veterans had lower PROMIS scores indicating less sleep disturbance and fatigue, and better global physical and mental health than younger veterans. Older veterans also reported better self-efficacy in managing symptoms and higher satisfaction with social roles and activities. However, mean PROMIS scores in all domains were in the moderate to severe range.

**Table 2:** Baseline diagnostic codes, pain and functional status characteristics in younger and older veterans enrolled in the VERDICT trial (*n* = 766)

	Age < 65 years *n* = 578	Age ≥ 65 years *n* = 188	Mean difference (95% CI)	*p* value
Low back pain ICD-10 codes n (%)^a^				*p* = .17
General	518 (89.6)	165 (87.8)		
Radicular	104 (18.0)	40 (21.3)		
Inflammatory	0	1 (0.5)		
Structural	25 (4.3)	12 (6.4)		
Pain duration > 5 years, n (%)	453 (78.4)	138 (73.4)		*p* = .16
High impact chronic pain, n (%)	373 (64.5)	117 (62.2)		*p* = .61
RMDQ score,^b^ mean (SD)	11.9 (5.2)	13.3 (4.9)	1.44 (0.59 to 2.28)	
PEG pain intensity,^c^ mean (SD)	6.2 (1.7)	5.9 (1.8)	− 0.24 (− 0.52 to 0.04)	
PEG overall score,^d^ mean (SD)	6.3 (1.7)	6.0 (1.9)	− 0.29 (− 0.58 to 0.01)	
PROMIS pain interference T-Score, mean (SD)	63.8 (4.8)	63.2 (5.0)	− 0.69 (− 1.49 to 0.11)	
PROMIS sleep disturbance T-score, mean (SD)	61.4 (7.6)	56.3 (8.5)	− 5.14 (− 6.44 to − 3.85)	
PROMIS fatigue T-score, mean (SD)	62.3 (7.8)	59.4 (7.1)	− 2.92 (− 4.18 to − 1.66)	
PROMIS satisfaction with roles and activities T-score, mean (SD)	59.4 (6.5)	57.7 (7.6)	− 1.70 (− 2.82 to − 0.58)	
PROMIS self−efficacy manage symptoms T-score, mean (SD)	59.9 (5.2)	57.9 (5.4)	− 1.97 (− 2.84 to − 1.10)	
PROMIS global physical health T-score, mean (SD)	63.0 (5.8)	61.9 (5.9)	− 1.18 (− 2.14 to − 0.22)	
PROMIS global mental health T-score, mean (SD)	60.7 (8.1)	55.6 (8.2)	− 5.14 (− 6.48 to − 3.80)	

*ICD-10* International Classification of Diseases, tenth revision, *RMDQ* Roland Morris Disability Questionnaire, *PEG*: pain, enjoyment, and general activity 3-item tool, *PROMIS* Patient-Reported Outcomes Measurement Information System

^a^General: non-specific/mechanical LBP; Radicular: radiculopathy, or inferred radiculopathy (e.g. stenosis); Inflammatory: inflammatory spondyloarthropathies; structural: diagnoses involving structural pathology and/or changes (e.g. spondylolysis, post laminectomy syndrome)

^b^Possible scores range from 0 (no disability) to 24 (extreme disability)

^c^Possible scores range from 0 (no pain) to 10 (pain as bad as you can imagine)

^d^Possible scores range from 0 (no pain or interference) to 10 (worst pain imaginable and complete interference)

### Substance use and mental health characteristics

Table 3 reports substance use and mental health characteristics among VERDICT participants at baseline, with older veterans reporting high levels yet lower prevalence of these health behaviors and co-morbidities than younger veterans. High-risk alcohol use was common in younger (25.4%) and older (18.1%) veterans (*p* = .05). Tobacco use also was substantial in both groups. Older veterans scored better on all self-reported mental health measures compared to younger veterans, including depression (31.4% vs. 44.8%, *p* = .001), anxiety (20.7% vs. 41.5%, *p* < .001), and PTSD (17.6% vs. 38.4%, *p* < .001). More younger veterans reported sleep disturbance than older veterans (57.1% vs. 34.6%, *p* < .001).

**Table 3:** Baseline substance use and mental health characteristics in younger and older veterans enrolled in the VERDICT trial (*n* = 766)

	Age < 65 years *n* = 578	Age ≥ 65 years *n* = 188	*p* value
High-risk alcohol use (AUDIT-C),^a^ n (%)	147 (25.4)	34 (18.1)	*p* = .05
Tobacco use, n (%)	100 (17.3)	26 (13.8)	*p* = .26
Depression (PHQ-2),^b^ n (%)	259 (44.8)	59 (31.4)	*p* = .001
Anxiety (GAD-7),^c^ n (%)	240 (41.5)	39 (20.7)	*p* < .001
Post-traumatic stress disorder (PTSD) (PCL-5),^d^ n (%)	222 (38.4)	33 (17.6)	*p* < .001
Sleep disturbance (PROMIS)^e^, n (%)	330 (57.1)	65 (34.6)	*p* < .001

*AUDIT* Alcohol Use Disorders Identification Test, *PHQ* Patient Health Questionnaire, *GAD* generalized anxiety disorder, *PCL* Posttraumatic Stress Disorder Checklist *PROMIS* Patient-Reported Outcomes Measurement Information System

^a^AUDIT-C is scored as the sum of the first 3 items of AUDIT. Possible scores range from 0 to 12. Score ≥ 4 in men or ≥ 3 in women indicates positive screen

^b^PHQ-2 is scored as the sum of the first two items of PHQ. Possible scores range from 0 to 6. Score ≥ 3 indicates positive screen

^c^Possible scores range from 0 to 21. Score ≥ 10 indicates a positive screen

^d^Possible scores range from 0 to 80. Score ≥ 33 indicates a positive screen

^e^Score ≥ 60 indicates a positive screen. PROMIS T-scores are constructed such that a referent population has mean 50 and SD 10. All PROMIS measures displayed here have been presented such that a higher score represents a worse outcome

#### Self-reported back pain treatments

Table 4 compares baseline self-reported back pain treatments used in the past 3 months. Younger (75.4%) and older (80.3%) veterans reported previous use of chiropractic care before enrolling in the VERDICT trial. Physical exercise, a recommended first-line treatment for LBP, was used as a treatment for pain, by only 16%. While two-thirds of the sample reported exercising in the past 3 months, older veterans were more likely to have received a clinician-provided recommendation to exercise. In contrast, the use of pharmacological treatment was widespread. Substantial proportions of both younger and older veterans used nonsteroidal anti-inflammatory drugs (NSAIDs, 62.8% vs. 56.9%, *p* = .18) or acetaminophen (49.4% vs. 63.3%, *p* < .001) for LBP. Older veterans reported higher use of gabapentin/neurontin (34% vs. 20.1%, *p* < .001) and spinal injections (18.6% vs. 13.2%, *p* = .06) than younger veterans. Younger veterans were higher users of muscle relaxants (31.7% vs. 17.6%, *p* < .001) and cannabis/marijuana (25.8% vs. 12.8%, *p* < .001) than older veterans, with use levels varying by site. Opioids (8.1 vs. 10.6%) and benzodiazepines (< 3.5%) were less common across age groups.

**Table 4:** Self-reported back pain treatments used at baseline by younger and older veterans enrolled in the VERDICT trial (*n* = 766)

	Younger Veterans Age < 65 years *n* = 578	Older Veterans Age ≥ 65 years *n* = 188	*p* value
Any previous use of chiropractic care	436 (75.4)	151 (80.3)	*p* = .17
Self-reported treatment use in the past 3 months, n (%)			
Tried physical exercise (any reason)	392 (67.8)	124 (66)	*p* = .64
Tried exercise to improve well-being or general health	288 (49.8)	89 (47.3)	*p* = .55
Tried exercise to manage pain	93 (16.1)	31 (16.5)	*p* = .90
Tried exercise to manage a symptom other than pain	8 (1.4)	3 (1.6)	*p* = .83
Source of exercise recommendation			
Used exercise on own	301 (52.1)	103 (54.8)	*p* = .52
Received exercise from a practitioner	62 (10.7)	32 (17)	*p* = .02
Medications			
Non-steroidal anti-inflammatory drugs (NSAIDs)	363 (62.8)	107 (56.9)	*p* = .18
Acetaminophen	285 (49.3)	119 (63.3)	*p* < .001
Gabapentin or Neurontin	116 (20.1)	64 (34.0)	*p* < .001
Muscle relaxants	183 (31.7)	33 (17.6)	*p* < .001
Cannabis/marijuana*	149 (25.8)	24 (12.8)	*p* < .001
Spinal injections	76 (13.2)	35 (18.6)	*p* = .06
Opioids	47 (8.1)	20 (10.6)	*p* = .28
Benzodiazepine	19 (3.3)	3 (1.6)	*p* = .23

*Cannabis use ranged by site: Connecticut (24%), Iowa City (22%), Los Angeles (30.4%), and Minneapolis (17.7%).

## Discussion

This cross-sectional study explored age differences in the baseline demographic and clinical characteristics of participants in a pragmatic, randomized trial of chiropractic care for veterans with CLBP. Our sample characteristics compared with national population estimates of U.S. veterans [49], in that VERDICT participants were predominately male and older. Veterans age ≥ 65 years made up 25% of our participants, which is similar to the percentage of self-reported chiropractic users in older non-veterans [50]. Both younger and older veterans reported a high prevalence of CLBP, long-standing and high-impact chronic pain, and moderate back-related disability, which is consistent with previous studies [4, 51, 52]. We also found potentially important differences in the presentation, daily impacts, and management of CLBP among veterans seeking chiropractic care in VA healthcare settings. The high percentages of younger veterans, female veterans, and veterans from diverse backgrounds enrolled in this trial reflect the changing demographics of patients seeking complementary and integrative care in military and veteran healthcare systems [53–55]. These differences in sociodemographic characteristics among veterans of different age cohorts will be explored in our planned primary and secondary analyses and may be informative for the broader LBP patient population in VA.

Outside of their differences in demographic backgrounds, other socioeconomic characteristics may impact on how trial participants engage in clinical care during the study. Younger and older veterans were similar in educational attainment, with most reporting some post-high school education, which is consistent with typical users of chiropractic care [56]. Overall, most veterans in this sample lived in non-rural settings (86%) and in neighborhoods with lower national ADI scores (75%). However, at the state level, between 45 and 49% of veterans in either age cohort in this study lived in settings with higher area deprivation index scores, which is linked with poorer access to food and health promoting behaviors, higher stress, more chronic disease, increased healthcare service utilization, and premature death [48, 57]. Further, more younger veterans reported potential employment challenges and lower partnered relationship statuses, which are associated with poorer health outcomes. Younger veterans also reported higher levels of 76–100% service-connected disability than older veterans. Service-connected disability in veterans is linked with higher mortality risk, lower socioeconomic status, and worse health outcomes compared with veterans without a service-connected disability and nonveterans [58]. Some VA healthcare users are also active National Guard or Reservists, making them subject to the time commitments of training exercises and/or deployments. These and other socioeconomic factors can present barriers to compliance with attending scheduled VA treatment visits and recommended active care instructions, which healthcare providers and patients should discuss when developing treatment plans [9, 10, 59, 60]. 

Older and younger veterans shared similar LBP ICD-10 diagnostic codes and pain profiles, such as the percentages of veterans reporting LBP duration of greater than 5 years and high-impact chronic pain. While some measures, such as pain intensity, pain interference, and back-related disability noted modest differences between age cohorts, these differences are unlikely to be clinically meaningful. For example, the 1.44-point difference in RMDQ scores between age groups is considered a slight difference (1–2) [61]. However, younger and older veterans differed on PROMIS scores and self-reported measures of mental health, substance use, sleep, fatigue, role satisfaction, and symptom self-management, with older adults reporting better scores, which may well be clinically significant. While many older veterans reported mental health concerns at baseline, more younger veterans scored highly on measures of depression, anxiety, and post-traumatic stress disorders. These differences in the burden of mental health concerns may influence attendance at study-related chiropractic treatment visits, which may in turn impact treatment outcomes. However, all veterans enrolled in VERDICT received usual care for their health conditions, including mental health services, as part of their overall VA healthcare. Our future analyses will report on medication use and concurrent healthcare visits, including those to mental health clinics, during the trial. From a clinical practice perspective, chiropractors treating veterans should be aware of the breadth of their pain-related and mental conditions, the ongoing management of both, as well as any possible unmet needs that might benefit from treatment or referral [9]. Importantly, the psychosocial challenges identified in the VERDICT sample underscore the potential of such initiatives as the 4Ms of Age-Friendly Care [62], the Geriatrics 5Ms [63], and the Whole Health model of care [64], which are frameworks designed to equip older adults and/or veterans of all ages with evidence-based self-care skills, health coaching, and clinician support to engage in personalized health plans. Future work should explore ways to better coordinate chiropractic care within these promising models of patient-centered, interprofessional healthcare delivery [65–67]. 

Higher percentages of older (18%) and younger (25%) veterans reported higher-risk alcohol use than the 6% to 8% reported in the VA Musculoskeletal Diagnosis Cohort in 2017 [4] or the monthly or less alcohol use reported by veterans in a pilot study conducted by our team before the COVID-19 pandemic [68]. Alcohol use was measured by the AUDIT-C, which is valid for both adult and older adult populations, and is used clinically for detecting unhealthy alcohol use in veteran populations [69]. However, AUDIT-C may underestimate higher-risk alcohol use, as the instrument does not capture the 40% larger standard U.S drink size, higher daily limits, or the lower recommended daily intake for older adults (1 drink per day for men and women) [70]. Veterans also reported substantial use of tobacco and cannabis compared to earlier evaluations [4]. One recent pilot survey of natural product consumption among 52 Veterans reported 40% used cannabis [71]. This exploratory study found differences in cannabis use by site (17.7% to 30.4%) that may warrant further investigation. The legal status and cultural acceptance of cannabis use varies historically by U.S. state. Cannabis use is fully legal in California (which was the first state to legalize medical marijuana in 1996) and is legal for recreational and medical use in Minnesota, Connecticut, and Illinois (which is geographically located near a satellite clinic for the Iowa site). However, in Iowa, cannabis use is restricted to limited medical use and is illegal for recreational use with first-offense possession punishable by fines and jail time in 2025. Cannabis use disorder has increased among veterans, particularly in jurisdictions that legalized its medical and recreational use [72]. Higher-risk alcohol use among veterans also increased since the COVID-19 pandemic [73]. Chiropractors working within a VA healthcare system can refer patients with substance use and mental health concerns to other VA healthcare providers via established referral mechanisms [9]. However, chiropractors working outside the VA setting are not confident in their ability to identify and refer patients misusing alcohol, drugs, or prescription medications [74]. Veterans also can experience barriers to seeking clinical care for substance use and mental health conditions, particularly those with exposure to trauma and warfare, depression, career concerns, preferences for self-care, and feeling unwelcomed or experiencing issues with wait times, costs, scheduling, childcare, and transportation in clinical settings [75], suggesting a need for ongoing training for chiropractors on these topics.

VA/DoD Clinical Practice Guideline recommends multimodal approaches to the treatment of LBP, including spinal manipulation and mobilization, physical activity, and pharmacological treatments [8]. Most veterans reported using chiropractic care in the past, and 67% had tried exercise, yet only 16% reported engaging in physical exercise for their pain in the previous 3 months. Unfortunately, the study instrument did not specify type of pain [76], so we cannot be certain whether VERDICT participants exercised as part of their back pain regimen. As physical exercise is a recommended first-line LBP management strategy [77], this low level of exercise engagement is concerning. Veterans may exercise for other reasons besides CLBP, although data about veteran exercise patterns is difficult to find. However, veterans with chronic musculoskeletal pain report higher pain intensity during and after exercise [78]. Chronic mental health problems and exposure to trauma also may keep veterans from exercising [79]. These findings reinforce the importance of individualizing chiropractic care with varied approaches for veterans of different age groups, employment status, comorbidities, and symptom burden. Chiropractors working with older adults might prioritize fall risk screening, consider gait and balance exercises, and coordinate management with primary care providers [9, 80]. Employed veterans might benefit from care plans that deliver work-based rehabilitation exercises and ergonomic counseling, along with SMT [81]. 

Veterans in both age groups used pharmacological treatments for CLBP management extensively, although often not in guideline-concordant patterns. NSAIDS were the most used medication in our sample and are recommended as a first-line treatment for CLBP, although with a notable small-to-moderate magnitude of effectiveness demonstrated for pain and no-to-small effect on physical function [61]. Other medications were used outside of current LBP guidelines. High numbers of veterans reported acetaminophen use. Recent placebo-controlled research indicates the medication is ineffective for acute back pain (such as experienced during a LBP flare-up) and has no evidence for its effectiveness in CLBP [82–85]. Opioids and benzodiazepines were used less often, which may be related to opioid safety initiatives in VA and research demonstrating users of chiropractic care are less likely to use opioids [49, 86]. The 4Ms of Age-Friendly Health Facilities can help guide chiropractors to identify patients who may be experiencing side effects from medications or polypharmacy as well as those patients whose pain levels are improving, either condition which may require a referral to the prescribing clinician [65]. 

In summary, our findings demonstrate the importance of raising awareness, improving accessibility, and better integrating chiropractic care into the veteran healthcare system, which aligns with VA guidelines promoting non-drug approaches to managing CLBP [87]. Given the significant population of veterans with CLBP within the VA healthcare system, enhancing access to non-pharmacological treatments like chiropractic care is urgent. Many of these veterans experience chronic pain that severely impacts their daily lives, and increasing access to chiropractic services can help reduce their reliance on opioids while improving pain management. Outside the VA healthcare system, insufficient knowledge about the benefits of chiropractic care, financial constraints, doubts about its efficacy and safety, racial and ethnic disparities, and accessibility issues—especially for those with mobility challenges or who live in rural areas—prohibit access to and use of chiropractic care services [88–90]. Within VA, chiropractic use is projected to continue increasing [12], yet veterans may still face barriers to optimal access and/or dosages [60, 91]. 

### Strengths and limitations

A major strength of this study was its large sample size (*n* = 766) of a diverse group of veterans from 4 VA geographically-disperse healthcare facilities, most of whom were seeking chiropractic care before trial enrollment and who report many characteristics to previous analyses of veterans with musculoskeletal conditions [4]. Our recruitment plan reached out to women veterans and veterans of all racial and ethnic backgrounds, which accounts for the higher percentages of these veterans compared to previous studies of patients receiving VA-based chiropractic care [18, 21]. VERDICT participants were likely to be representative of veterans with CLBP who are interested in receiving VA-based, on-station, chiropractic services and, perhaps, complementary and integrative health treatments [12, 92]. However, our sample may not represent veterans who prefer to receive chiropractic care outside the VA, are not interested in chiropractic, or seek alternative treatments, such as medication [93]. While datasets on veteran health are available publicly at the national or state levels [49, 94], data that allows comparison of veterans’ medical conditions and healthcare utilization among geographic sites smaller than the state-level, such as healthcare systems or facilities, are not readily available outside VA, which prevents us from determining how closely VERDICT participants match veterans seeking CLBP services in each study facility. However, previous research demonstrates that the clinical resources and approaches for CLBP pain management, including chiropractic care, do vary by VA setting, so not all veterans have similar access to the same treatments in each VA [18, 92, 95]. Further, VERDICT, like all clinical trials conducted with individual patients, is comprised of a self-selected sample of trial volunteers, who complete an informed consent process and additional health measures. These volunteers likely differ from veterans who do not enroll in clinical studies in some notable ways, such as comfort with online data collection systems and a willingness to follow treatment protocols [60], which introduces a level of non-representativeness or bias into our findings. Veterans with important variations in pain presentation (for example, shorter duration or higher levels of LBP intensity), demographics, or health beliefs may have opted out of the trial. Another strength is that VERDICT collected a robust battery of validated outcome measures which are harmonized with other PCTs aligned with the PMC [76]. However, by design, this secondary analysis does not compare patient-reported outcomes over time, which will be addressed in the primary analyses [22]. Self-reported data on previous treatment use, such as medications, has the potential for recall bias, which could be considered a limitation. As VA has robust clinical records for all participants, actual healthcare utilization will be verified and analyzed in subsequent papers. However, the types of pain and the clinical histories and breadth of treatments make any type of comparison very difficult. We expect substantial therapeutic variation according to medical and causal diagnoses, anatomic location, and treatment choices, which will be explored in other analyses. Finally, only 14% of participants resided in rural areas, which should be acknowledged as rural veterans may experience different healthcare access issues from those living in urban settings or near VA facilities [96]. 

## Conclusions

Among older and younger veterans seeking chiropractic care for CLBP in a pragmatic clinical trial, similar pain profiles were reported, but many age differences were noted in demographics, mental health and substance use, and CLBP treatments, which may impact patient-reported outcomes. Key demographics beyond age, such as sex, race, and ethnicity were not addressed here and will be the subject of future research.

## Supplementary Information

Below is the link to the electronic supplementary material.

Supplementary Material 1

Supplementary Material 2

## Data Availability

VERDICT metadata are posted on the NIH HEAL Initiative HEAL Data Platform at https://healdata.org/portal/discovery/HDP01154/. Demographics and participant-reported data will be available in a HEAL-compliant repository after 8/30/2026. Prior to that, the datasets used and/or analyzed for this paper are available from Dr. Long (long_c@palmer.edu) upon reasonable request.

## Abbreviations

ADI: Area deprivation index
AUDIT-C: Alcohol Use Disorders Identification Test-Concise
CLBP: Chronic low back pain
DOD: Department of Defense
DSM-5: Diagnostic and Statistical Manual of Mental Disorders, fifth edition
EHR: Electronic health record
GAD-7: General Anxiety Disorder-7
ICD-10: International Classification of Diseases, tenth revision
IRB: Institutional Review Board
LBP: Low back pain
MCID: Minimally clinically important difference
N: Number
NSAIDs: Nonsteroidal anti-inflammatory drugs
PCT: Pragmatic clinical trial
PEG: Pain, Enjoyment of life, and General activity
PHQ-2: Patient Health Questionnaire-2
PMC: Pain Management Collaboratory
PROMIS: Patient-Reported Outcomes Measurement Information System
PTSD PCL-5: Post-traumatic stress disorder checklist for DSM-5
REDCap: Research electronic data capture
RUCA: Rural–urban commuting area codes
RMDQ: Roland Morris Disability Questionnaire
SD: Standard deviation
STROBE: Strengthening the reporting of observational studies in epidemiology
U.S.: United States
VA: Veterans Affairs
VHA: Veterans Health Administration
VA CIRB: VA Central Institutional Review Board
VERDICT: Veterans response to dosage in chiropractic therapy
