# Supplementary material for: Age differences in demographic and clinical characteristics among veterans with chronic low back pain: a cross-sectional study of baseline findings from the Veteran Response to Dosage in Chiropractic Therapy (VERDICT) trial
Source: Chiropr Man Therap. 2025 Oct 13;33:44. doi: 10.1186/s12998-025-00613-z (PMC12516875; doi:10.1186/s12998-025-00613-z)
Supplement: Supplementary file 1 — Supplementary Material 1 [file 12998_2025_613_MOESM1_ESM.pdf]

Supplemental Material: Demographic Characteristics by Site

	Connecticut	Iowa City	Los Angeles	Minneapolis
Enrollment, n (%)	267 (34.9)	250 (32.6)	79 (10.3)	170 (22.2)
Age, mean (range)	49.3 (22-87)	50.6 (22-85)	47.1 (22-81)	57.1 (26-84)
Age <65 years, n (%)	208 (77.9)	197 (78.8)	68 (86.1)	105 (61.8)
Age ≥65 years, n (%)	59 (22.1)	53 (21.2)	11 (13.9)	65 (38.2)
Sex at birth, n (%)				
Male	222 (83.2)	190 (76.0)	64 (81.0)	127 (74.7)
Female	45 (16.9)	60 (24.0)	15 (19.0)	43 (25.3)
Race, n (%)				
White	173 (64.8)	197 (78.8)	26 (32.9)	136 (80.0)
Black or African American	59 (22.1)	23 (9.2)	33 (41.8)	16 (9.4)
Asian	2 (0.8)	0 (0.0)	7 (8.9)	3 (1.8)
American Indian or Alaskan Native	1 (0.4)	1 (0.4)	0 (0.0)	4 (2.4)
Native Hawaiian or Pacific Islander	4 (1.5)	1 (0.4)	0 (0.0)	3 (1.8)
Multiracial	1 (0.4)	6 (2.4)	1 (1.3)	0 (0.0)
Unknown or Not Reported	27 (10.1)	22 (8.8)	12 (15.2)	8 (4.7)
Ethnicity, Hispanic or Latino, n (%)	43 (16.1)	14 (5.6)	13 (16.5)	6 (3.5)
Highest Level of Education, n (%)				
Some grade school or high school	4 (1.5)	4 (1.6)	0 (0.0)	2 (1.2)
High school graduate	53 (19.9)	38 (15.2)	9 (11.4)	19 (11.2)
GED or equivalent	4 (1.5)	8 (3.2)	1 (1.3)	4 (2.4)
Some college or other program, no degree	68 (25.5)	72 (28.8)	27 (34.2)	40 (23.5)
Associate degree: occupational, technical, vocational, or academic	48 (18.0)	45 (18.0)	12 (15.2)	40 (23.5)
Bachelor’s degree (BA, AB, BS, BBA)	52 (19.5)	49 (19.6)	19 (24.1)	45 (26.5)
Master’s degree (MA, MS, MEng, MEd, MBA)	34 (12.7)	31 (12.4)	8 (10.1)	18 (10.6)
Professional degree (MD, DC, DDS, DO)	3 (1.1)	1 (0.4)	0 (0.0)	0 (0.0)
Doctoral degree (PhD, EdD)	1 (0.4)	2 (0.8)	3 (3.8)	1 (0.6)
Employment Status, n (%)				
Working for pay	141 (52.8)	125 (50.0)	34 (43.0)	72 (42.4)
Retired	65 (24.3)	73 (29.2)	18 (22.8)	73 (42.9)
Not currently employed	21 (7.9)	32 (12.8)	24 (30.4)	18 (10.6)
Taking care of house or family	6 (2.3)	1 (0.4)	1 (1.3)	1 (0.6)
Other	34 (12.7)	18 (7.2)	2 (2.5)	6 (3.5)
Relationship Status, n (%)				
Married or living with partner	145 (54.3)	127 (50.8)	27 (34.2)	88 (51.8)
Divorced or separated	61 (22.9)	58 (23.2)	18 (22.8)	43 (25.3)
Widowed	7 (2.6)	9 (3.6)	4 (5.1)	8 (4.7)
Never been married	54 (20.2)	56 (22.4)	30 (38.0)	31 (18.2)
Neighborhood Status, n (%)				
Rurality	16 (6.0)	66 (26.4)	3 (3.8)	23 (13.5)
