# Supplementary material for: Age differences in demographic and clinical characteristics among veterans with chronic low back pain: a cross-sectional study of baseline findings from the Veteran Response to Dosage in Chiropractic Therapy (VERDICT) trial
Source: Chiropr Man Therap. 2025 Oct 13;33:44. doi: 10.1186/s12998-025-00613-z (PMC12516875; doi:10.1186/s12998-025-00613-z)
Supplement: Supplementary file 2 — Supplementary Material 2 [file 12998_2025_613_MOESM2_ESM.pdf]

Supplemental Material: International Classification of Diseases, Edition 10 (ICD-10) Code for Low Back Pain

Q76.415	congenital kyphosis, thoracolumbar
Q76.426	congenital lordosis, lumbar
M43.9	deforming dorsopathy, unspecified
M43.26	fusion of spine, lumbar region
M43.27	fusion of spine, lumbosacral region
M43.28	fusion of spine, sacral and sacrococcygeal region
M43.25	fusion of spine, thoracolumbar region
M48.26	kissing spine, lumbar region
M48.27	kissing spine, lumbosacral region
M48.25	kissing spine, thoracolumbar region
G57.02	lesion of sciatic nerve, left lower limb
G57.01	lesion of sciatic nerve, right lower limb
G57.00	lesion of sciatic nerve, unspecified lower limb
M54.05	low back pain
M54.5	low back pain
M54.42	lumbago with sciatica, left side
M54.41	lumbago with sciatica, right side
M79.1	myofascial pain
M41.46	neuromuscular scoliosis, lumbar
M41.47	neuromuscular scoliosis, lumbosacral
M41.45	neuromuscular scoliosis, thoracolumbar
Q76.49	other congenital malformations spine, not assoc with scoliosis
M41.26	other idiopathic scoliosis, lumbar
M41.27	other idiopathic scoliosis, lumbosacral
M41.25	other idiopathic scoliosis, thoracolumbar
M51.36	other intervertebral disc degeneration, lumbar region
M51.37	other intervertebral disc degeneration, lumbosacral region
M51.26	other intervertebral disc displacement, lumbar region
M51.27	other intervertebral disc displacement, lumbosacral region
M51.25	other intervertebral disc displacement, thoracolumbar region
M53.86	other specified dorsopathies, lumbar region
M53.80	other specified dorsopathies, site unspecified
M47.896	other spondylosis, lumbar region
M47.897	other spondylosis, lumbosacral region
M43.8X6	other unspecified dorsopathies, lumbar region
M43.8X7	other unspecified dorsopathies, lumbosacral region
M43.8X8	other unspecified dorsopathies, sacral and sacrococcygeal region
M43.8X9	other unspecified dorsopathies, site unspecified
M43.8X5	other unspecified dorsopathies, thoracolumbar region
M54.00	panniculitis affecting regions of neck and back, site unspecffied
M54.16	radiculopathy, lumbar region
M54.17	radiculopathy, lumbosacral region
M53.3	sacrococcygeal disorder nec
M54.32	sciatica, left side
M54.31	sciatica, right side
M54.30	sciatica, unspecified side

M54.30	sciatica, unspecified side
M41.86	scoliosis (other), lumbar region
M41.87	scoliosis (other), lumbosacral region
M41.85	scoliosis (other), thoracolumbar region
M41.9	scoliosis (other), unspecified
M41.06	scoliosis, lumbar
M41.07	scoliosis, lumbosacral
M41.08	scoliosis, sacral and sacrococcygeal
M41.05	scoliosis, thoracolumbar
M99.03	segmental & somatic dysfunction, lumbar region
M99.05	segmental & somatic dysfunction, pelvic region
M99.04	segmental & somatic dysfunction, sacrum
M48.06	spinal stenosis, lumbar region
M48.07	spinal stenosis, lumbosacral region
M48.08	spinal stenosis, sacral and sacrococcygeal region
M48.05	spinal stenosis, thoracolumbar region
M43.16	spondylolisthesis, lumbar region
M43.17	spondylolisthesis, lumbosacral region
M43.19	spondylolisthesis, multiple sites in spine
M43.18	spondylolisthesis, sacral and sacrococcygeal region
M43.15	spondylolisthesis, thoracolumbar region
M43.06	spondylolysis, lumbar region
M43.07	spondylolysis, lumbosacral region
M43.09	spondylolysis, multiple sites in spine
M43.08	spondylolysis, sacral and sacrococcygeal region
M43.05	spondylolysis, thoracolumbar region
M47.16	spondylosis w myelopathy, lumbar region
M47.15	spondylosis w myelopathy, thoracolumbar region
M47.816	spondylosis w/out myelopathy or radiculopathy, lumbar region
M47.817	spondylosis w/out myelopathy or radiculopathy, lumbosacral region
S33.5xx	sprain of ligaments of lumbar spine
S33.8xx	sprain of other parts of lumbar spine and pelvis
S33.6xx	sprain of sacroiliac joint
S39.013	strain of muscle, fascia, and tendon of pelvis
S39.012	strain of muscle, fascia, and tendons of lower back
M99.13	subluxation complex (vertebral) of lumbar region

Reference: World Health Organization. ICD-10 : International Statistical Classification of Diseases and Related Health Problems: Tenth Revision. World Health Organization; 2004.
